# A sustainable nutritional solution for fattening pigs based on 25-hydroxycholecalciferol and triterpenoids added to a low Ca diet containing phytase improves growth performance via the activation of muscle protein synthesis without compromising bone mineralization

**DOI:** 10.1093/tas/txae152

**Published:** 2024-10-24

**Authors:** Estefania Pérez-Calvo, Ursula M McCormack, Ramon Muns, Christina Mulvenna, Laura Payling, Luis Romero, Laurent Roger, Maria C Walsh

**Affiliations:** Animal Nutrition and Health, DSM-Firmenich, Kaiseraugst, Switzerland; Animal Nutrition and Health, DSM-Firmenich, Kaiseraugst, Switzerland; Livestock Production Branch, Agri-Food and Biosciences Institute, Hillsborough, UK; Livestock Production Branch, Agri-Food and Biosciences Institute, Hillsborough, UK; Biofractal, Loulé, Portugal; Biofractal, Loulé, Portugal; Animal Nutrition and Health, DSM-Firmenich, La Garenne-Colombes, France; Animal Nutrition and Health, DSM-Firmenich, Kaiseraugst, Switzerland

**Keywords:** 25-hydroxycholecalciferol, bone mineralization, muscle, pigs, triterpenoids, vitamin D status

## Abstract

In the current climate of sustainable animal agriculture, nutritional strategies that support fattening swine growth performance and bone mineralization whilst reducing environmental impacts are much sought after. This study evaluated the effect of supplementing 25(OH)D_3_ with triterpenoids to a Ca-reduced diet containing phytase during the grower-finisher phase. Growth performance, bone composition, plasma metabolites and muscle gene expression were evaluated. Sixty crossbreed boar pigs (initial body weight (BW) 42.0 ± 5.1 kg at 12 wk of age) were assigned to three treatments with 20 pigs/treatment in a completely randomized design. Treatments comprised: 1) a standard commercial grower-finisher diet (positive control (PC)) containing 1,500 IU/kg vitamin D3 [3,585 kcal/kg digestible energy, 16.19% CP, 0.70% Ca, 0.29% standardized total tract digestible P]; 2) a negative control (NC) based on the PC with reduction in Ca and P (minus 30% and 10%, respectively); 3) the NC with vitamin D3 replaced by a commercially available compounds combination containing 25(OH)D3 and triterpenoids, dosed at 500 mg per kg of feed (TRT). All diets were provided ad libitum for 7 wk, and feed intake was recorded individually via electronic feeder stations. For the overall period, average daily gain and average daily feed intake were increased (P < 0.05) in TRT vs. NC or PC (+ 13.0% and + 8.3%, respectively, vs. NC); final BW was 7.8% higher vs. NC (+ 5.2% vs. PC; P < 0.05). Whole-body DXA-scanning at 19 wk of age showed that bone mineral density, content and percentage were reduced in NC vs. PC and equivalent to PC in TRT. Plasma 25(OH)D3 and P levels were raised in TRT (+ 33 ng/ml or 2.6-fold and + 0.55 mg/dL or 5.9%, respectively, vs. NC). The combination of 25(OH)D3 with triterpenoids was found to activate several biological pathways involved in muscle growth, including pathways that activate mTOR, a key central regulator of cell metabolism, growth, proliferation and survival when the gene expression was measured in the muscle tissue at 19 wk of age. These results suggest that the dietary combination of 25(OH)D3 with triterpenoids has the potential for use, alongside phytase, in supporting a reduction in Ca and P in the diet to reduce nutrient waste and improve the sustainability of production by promoting muscle growth and maintaining bone composition.

## Teaser

25-hydroxycholecalciferol and triterpenoids included in a low Ca diet increased growth performance in fattening pigs via the activation of muscle protein synthesis pathways without compromising bone mineralization.

## INTRODUCTION

Modern fattening swine production requires careful consideration of the nutritional requirements to meet the evolving needs of advanced genetics. These nutritional considerations apply not only to macro nutrients such as amino acids and energy but also to micronutrients such as minerals and vitamins. Mineral and vitamin availability must be sufficient to meet animal requirements but supplied at least-cost, with consideration for diet sustainability and minimizing waste. In growing-finishing pigs, dietary recommendations for Ca and P have been established to cover requirements for bone mineralization and lean muscle development (NRC, 2012).

As summarized in the NRC recommendations (NRC, 2012), there is a clear and close relationship between whole-body P mass and whole-body N mass, in which animal growth is optimized as dietary P increases. Hence, P is commonly supplemented as inorganic phosphate (iP) to ensure that available P is adequate. However, iP is costly, and any undigested P is excreted, which can accumulate in soil and contribute to environmental pollution via P runoff (Rothwell et al., 2022). Microbial phytases have been developed as highly efficacious nutritional solutions for improving the availability of P in feed raw materials and are used in most commercial pig diets today. Phytase can additionally increase the availability of Ca, energy, amino acids, and sodium in the diet (Cowieson et al., 2017).

Calcium is often over-supplied in commercial pig diets because limestone (the main Ca source) is a relatively inexpensive ingredient, and there is a lack of understanding about the actual (total) Ca content of the diet from all ingredient sources (Walk, 2016; Lagos et al., 2023). This is despite the knowledge that high dietary Ca can result in Ca functioning as an antinutrient which impairs the digestibility and absorption of P and retards animal growth (Stein et al., 2011; Hu et al., 2022). For example, an excess of only 0.20% Ca can result in a reduction in average daily gain (ADG) of at least 50 to 60 g per day (Merriman et al., 2017). Therefore, it is desirable to reduce dietary Ca levels closer to the requirement level, but this must be done carefully because if Ca is deficient or out of balance with P, Ca utilization will be redirected away from bone, resulting in bone weakness. Moreover, it is important to note that in growing-finishing pigs, although the dietary ratio of Ca:P needed to maximize bone mineralization increases linearly between 11 and 130 kg, that required to maximize growth performance decreases. The likely reason for this is that bone and soft tissue development are not constant during this period (Lautrou et al., 2021).

Vitamin D plays a key role in the absorption and retention of Ca and P. When Ca is low in the diet, the efficiency of Ca absorption is increased by its binding to 1,25-(OH)_2_D_3_, the metabolically active form of Vitamin D (González-Vega et al., 2016). To promote mineral absorption, vitamin D is supplemented in pigs, traditionally in the form of cholecalciferol (Vitamin D_3_). However, the supplementation of 25-hydroxycholecalciferol (25(OH)D_3_), which is one metabolic step closer than D_3_ to the active form, has been shown to increase circulating 25(OH)D_3_ levels more effectively than equivalent units of classical vitamin D3 (Lütke-Dörhoff et al., 2022). Vitamin D status is therefore important for efficient Ca absorption. Williams et al. (2024) reported that lame and unhealthy pigs showed lower circulating 25(OH)D_3_ levels than healthy pigs. The concentration of circulating 25(OH)D_3_ is accepted as the current standard for determining vitamin D status in swine (Arnold et al., 2015).

Moreover, recent research on Vitamin D supplementation has elucidated its importance for normal skeletal muscle development and optimizing muscle strength and performance (Iolascon et al., 2015). In pigs, the vitamin D status of sows appears to be crucial for the development of fetal skeletal muscle and has a consequent positive impact on growth performance and muscle development in the resulting piglets (Hines et al., 2013; Thayer et al., 2019; Zhang et al., 2019; Upadhaya et al., 2022). Studies evaluating the effect of vitamin D status on growth performance and muscle development in growing-finishing pigs are limited. In finishing pigs, Upadhaya et al. (2022) reported that diets supplemented with 25(OH)D_3_ (bioequivalent to 4,000 IU Vitamin D_3_/kg) exhibited increased circulating 25(OH)D_3_, faster weight gain, tended to exhibit increased feed efficiency and reduced expression of the MSTN gene (a negative regulator of muscle mass; Lee, 2004), compared to those fed only vitamin D_3_ at a lower dose level (2,000 IU/kg).

In the EU, either or both D_3_ and 25(OH)D_3_ can be supplemented up to a total level of 2,000 IU/kg feed, equivalent to 50 μ/kg (European Commission, 2019). This is below the level found to have a beneficial effect in finisher pigs on growth performance and muscle development in the study by Upadhaya et al. (2022). Therefore, there is a need to find nutritional strategies to boost the effect of vitamin D in this aspect. It has recently been documented that phytochemicals, such as triterpenoids, have significant antioxidant, antiinflammatory, and skeletal muscle atrophy protective effects (Wang et al., 2021; Rufino-Palomares et al., 2022; Yadav et al., 2022). Thus, in the present study, it was hypothesized that the combination of dietary 25(OH)D_3_ and triterpenoids, which would provide a further enhancement to growth performance in a synergistic way with 25(OH)D_3_, via increasing muscle development. The aim was to establish whether the dietary combination of 25(OH)D_3_ with triterpenoids, when added to a low Ca, P-adequate diet could improve growth performance to a level similar to that achieved by an unsupplemented diet containing a control level of Ca (above or below NRC, 2012 recommendations) and P (at NRC requirements), classical vitamin D_3_ at 1,500 IU/kg and with phytase in the background, without compromising bone mineralization. Alongside growth performance and bone mineralization measurements, a transcriptomics approach was used to evaluate the expression of genes associated with muscle tissue growth and other key biological pathways to gain insight into the underlying mechanisms of the effect of the dietary combination of 25(OH)D_3_ with triterpenoids.

## MATERIALS AND METHODS

The study was conducted at the Agri-Food and Biosciences Institute (AFBI), Hillsborough, Northern Ireland, UK. The research was carried out under Project License Number PPL2912 in accordance with the Animals (Scientific Procedures) Act 1986 (The Parliament of the United Kingdom, 1986).

### Animals, Diets and Experimental Design

Sixty boar pigs [Duroc × (Landrace × Large White), initial body weight (BW) 30.0 ± 3.9 kg, 10 wk of age] were obtained from the AFBI Hillsborough research herd, group-housed (10 pigs/pen) in an experimental finishing house and offered a commercial grower diet [3,585 kcal/kg digestible energy (DE), 17.5% crude protein (CP), 1.0% dig Lys, 0.65% Ca, 0.45% P, and Vitamin D_3_ at 2,000 IU/kg] ad libitum until 12 wk of age. At 12 wk of age (BW 42.0 ± 5.1 kg), pigs were assigned to receive one of three experimental diets (2 pens/treatment, balanced for BW) in a completely randomized design. The experimental diets comprised: 1) a positive control (PC) comprising a standard commercial grower-finisher diet formulated to contain 3,346 kcal/kg DE, 16.19% CP, 0.95% digestible Lys, 2.94% crude fiber (CF), 0.70% Ca (equivalent to 120% of the NRC, 2012 recommendation) and 0.29% standardized total tract digestible (STTD) P (approximately equal to the NRC, 2012 recommendation), supplemented with 1,500 IU/kg feed of Vitamin D_3_ (Rovimix D_3_-500; DSM-Firmenich, Switzerland); 2) a negative control (NC), based on the PC but containing 0.49% Ca (below the NRC, 2012 recommendation) and 0.26% STTD P (approximately equal to the NRC, 2012 recommendation but lower than PC), supplemented with 1,500 IU/kg of Vitamin D_3_ (Rovimix D_3_-500; DSM-Firmenich, Switzerland) as in the PC and; 3) the NC diet but with the cholecalciferol replaced by a commercially available combination (MaxiFicient Boost GF, DSM-Firmenich, Switzerland) comprising 1,500 IU/kg of 25-hydroxycholecalciferol (25(OH)D_3_), Rovimix HyD 1.25%, (DSM-Firmenich, Switzerland) and triterpenoids (TRT). All diets contained 1,000 FYT/kg of a supplemental phytase produced in *Aspergillus oryzae* (Ronozyme HiPhos, DSM-Firmenich, Switzerland), added as part of the vitamin-mineral premix. Diets and water were provided ad libitum until the end of the study at 19 wk of age. Diets were sampled for the analysis of nutrients, energy, and Vitamin D_3_.

### Growth Performance Measurements

Pigs were monitored daily for health, and any dead animals were removed and individually weighed. Feed intake was monitored daily on an individual pig basis by electronic feeders. Each pen was equipped with a Feed Information Recordable Equipment station, which contained an on-board electronic digital Animal Weighing platform and digital Feed Trough Weighing system (FTW) (Compident MLP Pro Schauer, Prambachkirchen, Germany), which recorded the feed intake, BW, and the identity of each pig (from ear tags) upon entry to the feeding station. Pig BW was measured individually at 12, 16, and 19 wk of age. Average daily feed intake (ADFI), average daily gain (ADG) and feed conversion ratio (FCR) were calculated on an individual basis for the following periods: 12–16, 16–19, and 12–19 wk of age.

### Body and Forelimb Composition

Whole-body composition scanning was performed at 11 and 19 wk of age on 24 pigs (8 pigs per treatment) selected at random. At 11 wk of age, selected pigs were weighed, transferred to a sedation pen, sedated according to weight and scanned using a Dual-energy X-Ray absorptiometry (DXA) scanner as described by Hawe et al. (2020). Sedation comprised two separate intramuscular injections administered via the neck, first of 0.5 mL/10 kg Stresnil (Elanco Ltd, Basingstoke, UK) and then of 2 mL/10 kg Ketamidor (Chanelle Pharma Ltd, Galway, Ireland). Sedated pigs were transported to the DXA scanner and underwent a whole-body composition scan, and a separate scan of the front left forelimb. After the scan, animals were transported to individual recovery pens for a minimum of 4 h. After complete recovery, animals were reintroduced to their original experimental pen and monitored for a further 4 h to ensure no adverse effects.

At the end of the experiment (19 wk of age), all animals were sedated using the same previous protocol and whole-body and left forelimb scans were performed as before within 10 min of euthanasia using 15 mL of intracardiac injection of pentobarbital (Dolethal, Vetoquinol, Paris, France). Scans were assessed using a Stratos DR device equipped with the Stratos DR (v4.0.7.0 11–16–2016) software package (Mi Healthcare, Knowsley, UK). Images were analyzed in accordance with the methodologies described by Kipper et al. (2018); total bone mineral density (BMD), bone mineral content (BMC), total mass and percentage of bone, tissue, lean, and fat were calculated.

### Blood, Bones, and Tissue Sampling

Blood samples (10 mL) were obtained from all pigs at 19 wk of age by venipuncture of the jugular. Samples were centrifuged at 3,000 rpm for 15 min to obtain plasma which was stored at -80°C.

After euthanasia (within 20 min), 16 pigs/treatment were dissected to obtain a 0.5 cm^3^ sample of the longissimus dorsi muscle. Samples were rinsed with ice-cold PBS, placed in Eppendorf tubes containing RNAlater solution and stored at −80 °C prior to analysis. In addition, 12 pigs/treatment were sampled for metatarsal bones collected from the front left forelimb. These were removed, and the third metacarpal dissected and stored in a freezer (−20 °C) for later measurements.

### Chemical Analysis

Diet samples were analyzed in duplicate for dry matter (DM), ash, N, gross energy, Ca, P, lipids, and CF, according to validated methods (VDLUFA, 1976; AOAC, 2006). The percentage of DM and ash was calculated by the mass difference after oven drying for 24 h at 100 °C and 12 h at 600 °C, respectively. Nitrogen concentration was analyzed using the Dumas combustion method using an automatic N analyzer (TruMac N; LECO Corp., St. Joseph, MI), and CP was calculated as N × 6.25. Gross energy is determined using an adiabatic bomb calorimeter (C 2000 basic, IKA, Staufen, Germany). Lipid analysis was conducted by Larebron (Illkirch, France) by hot acidic etching with HCl, followed by continuous extraction with petroleum ether and determination via a gravimetric method. CF was analyzed by hot extraction with sulfuric acid and potassium hydroxide at 550 °C in an automated Fibersac A2000 (Ankom Technology), followed by cold extraction with acetone.

Metacarpal bone samples were de-fleshed, cartilaginous caps were removed, and 2.2 cm of the middle section of the bone was extracted. These sections were used to assess bone ash percentage, which was expressed as the percentage of dry bone weight after drying at 105 °C for 24 h followed by incineration at 550 °C for 48 h.

Diets and bone samples were analyzed for Ca and P by Inductively Coupled Plasma-Optical Emission Spectrometry (ICP-OES, 5100 Dual View, Agilent) using an in-house method based on DIN EN ISO 11885:2007 (AOAC, 2006) after mineralization in 18.5% HCl. Calcifediol (vitamin D_3_) and calcidiol (25(OH)D_3_) in diet samples were analyzed. Vitamin D_3_ was analyzed by adding an internal standard, followed by saponification of the sample with potassium hydroxide alkaline ethanol solution and extraction with cyclohexane. Quantification was by reversed-phase HPLC-MS/MS using 6,19,19-trideutero-vitamin D_3_ as an internal standard. Calcidiol (25(OH)D_3_) was analyzed by adding an internal standard, saponifying the sample and extracting it with tert-butyl methyl ether. Extracted samples were dried by evaporation, solubilized, and quantified by reversed-phase HPLC-MS/MS using labeled deuterium-6–25-hydroxy cholecalciferol as an internal standard. One phytase unit (FYT) was defined as the amount of enzyme that released 1 µmol of inorganic phosphate from 50 mM phytate per minute at 37 °C and pH 5.5. These analyses were performed in duplicate, except that phytase activity in the feed samples was determined from three replicates.

Plasma samples were analyzed for 25(OH)D_3_, Ca, P, alkaline phosphatase (ALP), creatinine, urea, total protein, albumin, C-terminal telopeptide (CTX), osteocalcin, and IGF1. Vitamin D_3_ and 25(OH)D_3_ were analyzed by the addition of an internal standard to an aliquot of plasma, followed by extraction by protein precipitation with acetonitrile. After centrifugation and filtration, the supernatant was evaporated, and the residue was reconstituted in methanol-acetonitrile-water solution. Analysis was performed by reversed-phase LC-MS. Alkaline phosphatase, Ca, P, urea, total protein, creatinine, and albumin were determined using a Biomedical automated COBAS 6000 (Roche Diagnostics, CH-4202 Basel) using the respective Roche Diagnostic kits. Osteocalcin and CTX were determined by ELISA. Samples were diluted in the provided dilution buffer of the ELISA kits, 1:2 (CTX) and 1:5 (osteocalcin) and analyzed by -MID Osteocalcin ELISA (AC-11F1; Immunodiagnostic Systems) and Serum CrossLaps CTX-I ELISA (AC-02F; Immunodiagnostic Systems), respectively, after testing for cross-reactivity in pigs. For the analysis of IGF1, samples were diluted 1:100 and measured with the Quantikine ELISA, human IGF-I/IGF-1 Immunoassay (SG100B + DG100B; R&D Systems).

### Gene Expression Analysis in Muscle Tissue

Total RNA was extracted from muscle tissue samples using Trizol reagents (Invitrogen, Carlsbad, CA) and purified with RNeasy mini kits (Qiagen, Valencia, CA) according to the manufacturer’s instructions. Extracted RNA was suspended in RNase-free water, and the sample purity and concentration were measured on an Agilent 2100 Bioanalyzer (Agilent Technologies, Santa Clara, CA). The integrity of the extracted RNA (RIN) was assessed by a Bioanalyzer (Agilent Technologies, Palo Alto, CA) and found to range from 4 to 8.2. Data with a RIN less than 6 were excluded, resulting in 14, 13, and 15 replicate samples for the PC, NC, and TRT groups, respectively, and a mean RIN of 7. Extracted RNA was used to prepare cDNA libraries using a NEBNext Ultra RNA Library Prep Kit for Illumina and sequenced with 150 bp paired-end reads on the Novaseq 6000 Illumina platform. Sequencing generated 20 to 30 M reads per sample. FastQC (version 0.11.9) was used to quality-check raw reads. Data were cleaned using TrimGalore (RRID:SCR_011847) to remove poor-quality portions of the reads and unwanted sequences, such as poly-A tails or adapters, as described by Martin (2011). Pseudo-alignment was conducted using Kallisto whereby short sequences of bases (k-mers) were mapped to the reference transcripts, and De Bruijn graphs were used to align overlapping sequences, as previously described by Bray et al. (2016). Counts (the number of sequences assigned to each gene) were generated through alignment to the *Sus Scrofa* reference genome (version Sscrofa 11.1).

### Statistical Analysis

Growth performance data, plasma metabolites, bone measures, and body and carcass composition data were all analyzed by one-way ANOVA, including treatment as a main effect. The pig was the experimental unit for these analyses. Initial BW (at 12 wk of age) was included as a covariate for the growth performance analysis. For body composition analysis BW at 11 wk of age was included as a covariate for the analysis of 11 wk whole-body and forelimb scan data, and BW at 19 wk of age was included as a covariate for the analysis of the 19 wk final scan data. Means separation was achieved using Fisher’s Least significant Difference test. Statistical analyses were carried out using Genstat version 21.1. A *P*-value of < 0.05 was considered to be statistically significant. 0.05 < *P* < 0.1 was considered as a tendency.

Gene expression data were normalized using DeSeq2 (as described by Love et al., 2014) to account for technical variation due to differences in sequencing depth and mRNA composition among samples. Differential gene expression among samples was used to quantify and statistically analyze the effect of the different treatments on biological pathways in muscle tissue. Using DeSeq2, genes were ranked based on an estimation of the logarithmic fold change in their expression. The significance of these differential expressions was then tested with reference to biologically relevant thresholds. DeSeq2 generates a *P-*value from a Wald test, which is based on the production of a *Z*-score and then adjusts this using the Benjamini and Hochberg method (Benjamini and Hochberg, 1995) to correct for multiple comparison testing and the corresponding false discovery rate. An adjusted *P-*value of < 0.10 was selected as the statistical significance threshold for identifying differentially expressed genes (DEG).

A topology-based Quantitative Pathway Activation method (QPA) developed by Biofractal (Portugal), based on statistically powerful methods for network enrichment analysis identified by Ma et al. (2019), was used to identify biological pathways of interest from DEG among the treatments. The QPA method uses the expression levels of genes in the pathway, their statistical significance and their topological importance to generate a pathway activation score. The activation score represents the number of standard deviations a given data point lies above (positive score) or below (negative score) the control mean. The pathway catalog was based on Reactome (Gillespie et al., 2022) with customizations from Gene Ontology (Ashburner et al., 2000). Treatment was included as a main effect and RIN as a covariate in the statistical models for DEG and QPA analysis.

## RESULTS

### Diet Analysis

The analyzed nutrient and energy content of the treatment diets is presented in Table 1. Analyzed CP levels were two percentage points lower than expected in all diets, whereas analyzed Ca and P levels matched well with formulated values and were similar in the NC and TRT diets but lower in these treatments than in the PC (by 24–28% and 11–14%, respectively, for Ca and P), as intended. Concentrations of Vitamin D_3_ and 25(OH)D_3_ were slightly lower than expected in all diets (by 8.5–15%) but within acceptable limits. The analyses verified the presence of Vitamin D_3_ in NC and PC and of 25(OH)D_3_ in TRT. Analyzed phytase activity was variable but within acceptable limits to confirm its supplementation.

**Table 1.** Ingredient and nutrient composition of the experimental diets

Item	PC	NC	TRT^*a*^
Ingredient, g/kg (as-fed basis)			
Barley	250	250	250
Wheat	260	272	272
Corn	250	250	250
Soya bean meal	186	184	184
Soya oil	12.0	9.00	9.00
Limestone	12.0	8.00	8.00
Mono dicalcium phosphate	1.79	0.00	0.00
Salt	4.61	4.62	4.62
L-Lysine HCl	2.12	2.15	2.15
DL-methionine	0.53	0.51	0.51
L-threonine	0.34	0.34	0.34
Vitamin-mineral premix 1^*b*^	5.00	5.00	0.00
Vitamin-mineral premix 2^*c*^	0.00	0.00	5.00
MaxiFicient Boost GF^*d*^	0.00	0.00	0.50
Evobase^*e*^	15.0	15.0	15.0
Calculated nutrient composition			
Digestible energy, kcal/kg	3,346	3,346	3,346
Ash, %	4.56	3.95	3.95
Crude protein, %	16.19	16.19	16.19
Crude fat, %	3.14	2.84	2.84
Crude fiber, %	2.94	2.96	2.96
Lysine, %	1.05	1.05	1.05
Calcium, %	0.70	0.49	0.49
STTD phosphorus, %	0.29	0.26	0.26
SID lysine, %	0.95	0.95	0.95
SID methionine, %	0.32	0.32	0.32
SID methionine + cystine, %	0.56	0.56	0.56
SID threonine, %	0.60	0.60	0.60
SID tryptophan, %	0.17	0.17	0.17
Vitamin D_3_, IU/kg	1,500	1,500	0
25(OH)D_3_, IU/kg	0.00	0.00	1,500
Analyzed composition			
Gross energy, kcal/kg	3,899	3,897	3,893
Dry matter, %	88.1	87.7	87.7
Ash, %	4.40	3.80	3.80
Crude protein, %	14.7	14.4	14.6
Crude fat, %	4.60	3.80	4.60
Crude fiber, %	3.00	2.90	3.10
Calcium, %	0.71	0.54	0.51
Phosphorus, %	0.37	0.33	0.32
Vitamin D_3_, IU/kg	1,270	1,350	<LOD
25(OH)D_3_, IU/kg	<LOD	<LOD	1,372

^*a*^TRT = NC + Maxificient Boost GF, a combination of 25(OH)D_3_ plus triterpenoids (500 mg/kg; MaxiFicient Boost GF, DSM-Firmenich, Switzerland corresponding to 1,500 IU/kg vitamin D).

^*b*^Containing, per kilogram of feed: vitamin A, 6,510 IU; vitamin D, 1,500 IU; vitamin E, 100 mg; vitamin K3, 0.46 mg; vitamin B1, 0.79 mg; vitamin B2, 6.0 mg; pantothenic acid, 17.8 mg; vitamin B6, 1.6 mg; vitamin B12, 30 mg; nicotinic acid, 20 mg; folic acid, 0.51 mg; biotin, 99.9 μg; choline, 99 mg; iron, 100 mg; copper, 15 mg; manganese, 40 mg; zinc, 65 mg; iodine, 2 mg; selenium, 0.4 mg; phytase, 1,000 FYT; citric acid, 17.5 mg; butylated hydroxtoluene, 52.1 mg; propyl gallate, 27.3 mg; butylated hydroxyanisole, 34.6 mg; sepiolite, 500 mg; silicic acid, 112 mg, and 1,000 FYT phytase (Ronozyme HiPhos; DSM-Firmenich, Switzerland).

^*c*^Containing the same chemical composition as vitamin-mineral premix 1 except without vitamin D.

^*d*^Maxificient Boost GF (DSM-Firmenich, Switzerland) is a combination of 25(OH)D_3_ added at 0.04 mg/kg corresponding to 1,500 IU/kg vitamin D, plus triterpenoids and a carrier.

^*e*^EvoBase is a source of sugar-bound amino acids which are slowly released in the small intestine to optimize absorption (Devenish Ltd., Belfast, UK).

IU, international units; LOD, limit of detection; SID, standardized ileal digestible.

### Growth Performance

The effect of treatment on growth performance is presented in Table 2. At the start of the experiment (12 wk of age), there was no difference between treatments in BW, ADG, ADFI or FCR (*P* > 0.05). There were no effects of treatment on response measures during 12–16 wk of age. During 16–19 wk of age, ADG and ADFI did not differ in NC vs. PC, but both were increased (*P* < 0.05) in TRT compared to NC or PC (+20.9% and +12.4% vs. NC, respectively); BW at 16 wk of age was similar across all treatments. For the overall period (12–19 wk of age), again ADG and ADFI did not differ in NC vs. PC, but both were increased in TRT (by 13.0% and 8.3%, respectively, vs. NC). Final BW at 19 wk of age was also increased in TRT vs. NC or PC (+7.8 and +5.2%, respectively; *P* < 0.05). There was no statistically significant impact of treatment on FCR during the experimental period.

**Table 2.** Effect of treatment on BW, ADG, average daily feed intake (ADFI) and FCR^*a*^

Item	PC	NC	TRT^*b*^	SEM	*P*-value
BW, kg/pig					
12 wk of age	43.87	41.83	40.19	1.624	0.090
16 wk of age	71.61	70.82	72.40	1.321	0.502
19 wk of age	102.89b	100.45b	108.26a	2.368	0.007
ADG, kg/pig					
12–16 wk of age	1.06	1.03	1.09	0.047	0.502
16–19 wk of age	1.36b	1.29b	1.56a	0.072	0.002
12–19 wk of age	1.19b	1.15b	1.30a	0.046	0.007
ADFI, kg/pig					
12–16 wk of age	2.01	1.99	2.06	0.062	0.502
16–19 wk of age	2.98b	2.90b	3.26a	0.105	0.003
12–19 wk of age	2.50b	2.40b	2.60a	0.075	0.027
FCR					
12–16 wk of age	1.91	1.95	1.91	0.066	0.788
16–19 wk of age	2.22	2.28	2.10	0.084	0.114
12–19 wk of age	2.05	2.11	2.00	0.050	0.130

^*a*^Values are the means of each treatment.

^*b*^TRT = NC + Maxificient Boost GF, a combination of 25(OH)D_3_ plus triterpenoids (500 mg/kg; MaxiFicient Boost GF, DSM-Firmenich, Switzerland corresponding to 1,500 IU/kg vitamin D).

a,bMeans without a common superscript are significantly different at *P *< 0.05.

### Plasma Metabolites

The effect of treatment on plasma metabolites at 19 wk of age is shown in Table 3. Vitamin D_3_ concentrations were not significantly different in NC vs. PC (*P* > 0.05) and below the limit of quantification in TRT. However, 25(OH)D_3_ concentrations were similar in PC and NC but increased in TRT (+34 ng/mL and + 33 ng/mL vs. NC and PC, respectively; *P* < 0.05). Levels of 24, 25-(OH)_2_-D_3_ were also higher in TRT vs. NC or PC (+10.23 and + 10.40 ng/mL, respectively; *P* < 0.05). Plasma Ca concentrations did not differ among treatments, but plasma P was increased in TRT vs. NC or PC (+ 0.55 mg/dL or 5.9% vs. NC and + 0.66 mg/dL or 7.2%, respectively; *P* < 0.05). In addition, CTX was increased in TRT vs. NC (+26.9%; *P* < 0.05) but was equivalent to PC. Other measured metabolites did not differ among treatments.

**Table 3.** Effect of treatment on plasma metabolites at 19 wk of age^*a*^

Item	PC	NC	TRT^*b*^	SEM	*P*-value
Vitamin D_3_, ng/mL^*c*^	4.70a	5.10a	<0.50b	0.200	<0.001
25(OH)D_3_, ng/mL	19.10b	20.14b	53.10a	1.341	<0.001
24, 25-(OH)_2_-D_3_, ng/ml	3.01b	3.18b	13.41a	0.384	<0.001
Ca, mg/dL	11.25	11.07	10.94	0.108	0.144
P, mg/dL	9.14b	9.25b	9.80a	0.144	0.007
CTX, ng/mL^*d*^	0.31ab	0.26b	0.33a	0.018	0.030
Osteoclastin, ng/mL	91.21	100.52	98.09	5.171	0.409
Alkaline phosphatase, U/L	210.56	213.07	218.49	20.357	0.946
Urea, mg/dL	17.73	19.54	17.34	1.144	0.370
Total protein, mg/dL	65.18	65.02	64.11	0.860	0.670
Creatinine, mg/dL	1.23	1.27	1.27	0.026	0.546
Albumin, mg/dL	4.27	4.22	4.33	0.077	0.632
IGF-1, ng/mL^*e*^	391.09	365.69	360.42	10.513	0.107

^*a*^Values are the means of each treatment.

^*b*^TRT = NC + Maxificient Boost GF, a combination of 25(OH)D_3_ plus triterpenoids (500 mg/kg; MaxiFicient Boost GF, DSM-Firmenich, Switzerland corresponding at 1,500 IU vitamin D3/kg feed).

^*c*^A value of < 0.5 means less than the limit of quantification.

^*d*^CTX = C-terminal telopeptide.

^*e*^IGF1 = Insulin-like growth factor 1.

a,bMeans without a common superscript are significantly different at *P *< 0.05.

### Body and Bone Composition

The effect of treatment on whole-body bone, lean, fat, and tissue mass composition, as well as on metacarpal mineralization, is presented in Table 4. There were no differences in these measures among treatments at the start of the experiment (12 wk of age). At 19 wk of age, BMD, BMC, and bone percentage were all reduced in NC vs. PC (−4.9%, −3.5%, and −0.08% points, respectively; *P* < 0.05), whereas tissue percentage was increased (+0.08% points; *P* < 0.05). Pigs fed TRT exhibited BMD, BMC, bone percentage, and tissue percentage responses that were numerically intermediate between NC and PC but not significantly different from PC or NC. Metacarpal bone ash, Ca, and P content at 19 wk of age were all reduced in NC vs. PC when results were expressed in g/bone (−10.7%, −10.8%, and −10.4% points, respectively, *P *< 0.05) but did not differ when results were expressed in percentage terms. The metacarpal bone response of pigs fed TRT was numerically in between that of pigs in the NC and PC when expressed in g/bone.

**Table 4.** Effect of treatment on whole-body bone, lean, fat, and tissue mass composition at 11 and 19 wk of age and on metacarpal content at 19 wk of age^*a*^

Item	PC	NC	TRT^*b*^	SEM	*P*-value
Bone mineral density, g/cm^2^					
11 wk of age	0.81	0.86	0.82	0.024	0.198
19 wk of age	1.03a	0.98b	1.01ab	0.020	0.030
Bone mineral content, g/pig					
11 wk of age	896	1088	896	128.1	0.318
19 wk of age	2,294a	2,214b	2,227ab	31.3	0.030
Lean mass, kg/pig					
11 wk of age	25.6	34.7	26.2	5.85	0.312
19 wk of age	86.1	86.3	85.6	1.28	0.890
Fat mass, kg/pig					
11 wk of age	7.57	9.29	7.85	1.066	0.315
19 wk of age	22.5	23.2	24.0	0.91	0.298
Tissue mass, kg/pig					
11 wk of age	33.2	44.0	34.1	6.89	0.309
19 wk of age	108.6	109.5	109.6	1.08	0.590
Bone, %					
11 wk of age	2.63	2.52	2.57	0.080	0.425
19 wk of age	2.07a	1.99b	2.00ab	0.030	0.020
Lean, %					
11 wk of age	75.1	75.9	75.0	0.90	0.641
19 wk of age	77.7	77.3	76.6	0.85	0.500
Fat, %					
11 wk of age	22.2	21.6	22.4	0.88	0.674
19 wk of age	20.3	20.7	21.4	0.85	0.450
Tissue, %					
11 wk of age	97.4	97.5	97.4	0.080	0.425
19 wk of age	97.9b	98.1a	98.0ab	0.030	0.020
Metacarpal content at 19 wk of age, g/bone					
Bone ash	1.84a	1.65b	1.72b	0.045	0.014
Bone Ca	0.69a	0.62b	0.64b	0.017	0.014
Bone P	0.33a	0.29b	0.31ab	0.008	0.019
Metacarpal content at 19 wk of age, %					
Bone ash	58.1	56.3	58.2	0.840	0.220
Bone Ca	21.7	21.0	21.6	0.323	0.284
Bone P	10.3	10.0	10.4	0.152	0.228

^*a*^Values are the means of each treatment.

^*b*^TRT = NC + Maxificient Boost GF, a combination of 25(OH)D_3_ plus triterpenoids (500 mg/kg; MaxiFicient Boost GF, DSM-Firmenich, Switzerland corresponding to 1,500 IU/kg vitamin D).

a,bMeans without a common superscript are significantly different at *P *< 0.05.

### Gene Expression and Biological Pathway Activation in Muscle

**Figure 1.**
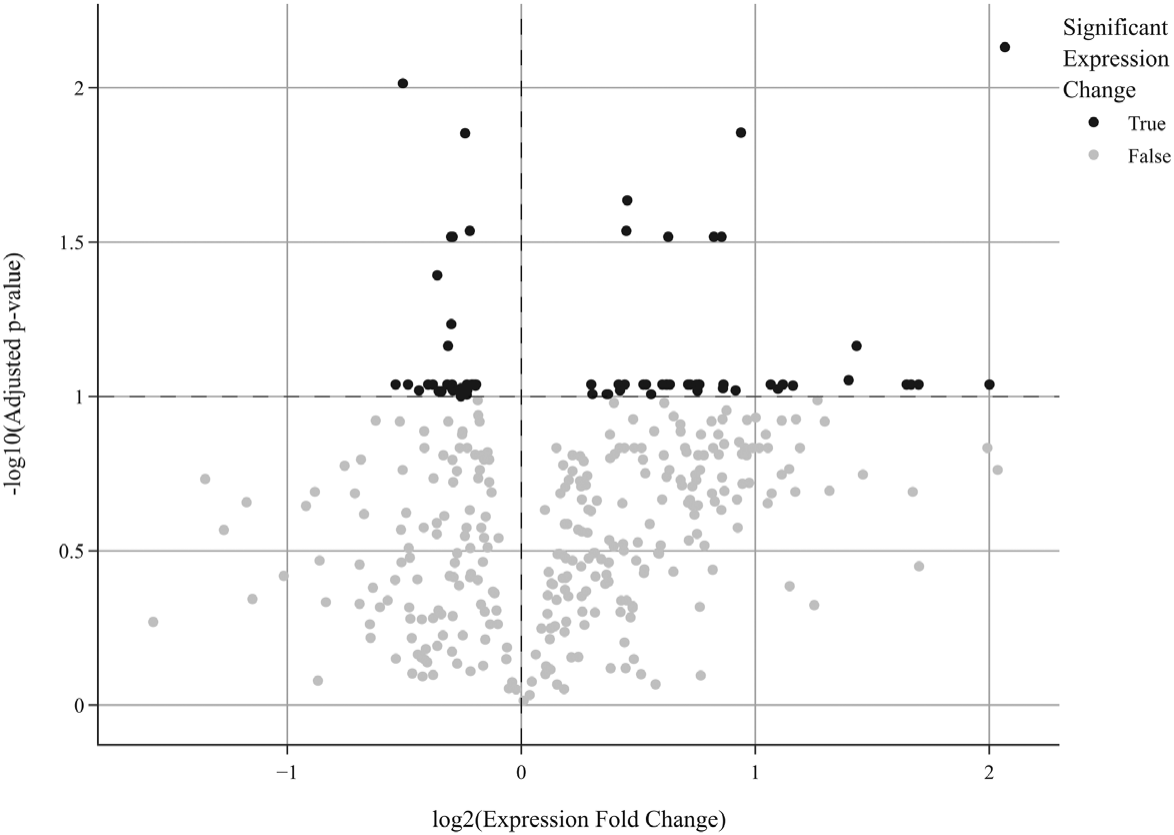
Volcano plot highlighting DEG (identified by black dots) in the muscle tissue of PC vs. NC pigs (71 DEG; adjusted *P* < 0.1).

**Figure 2.**
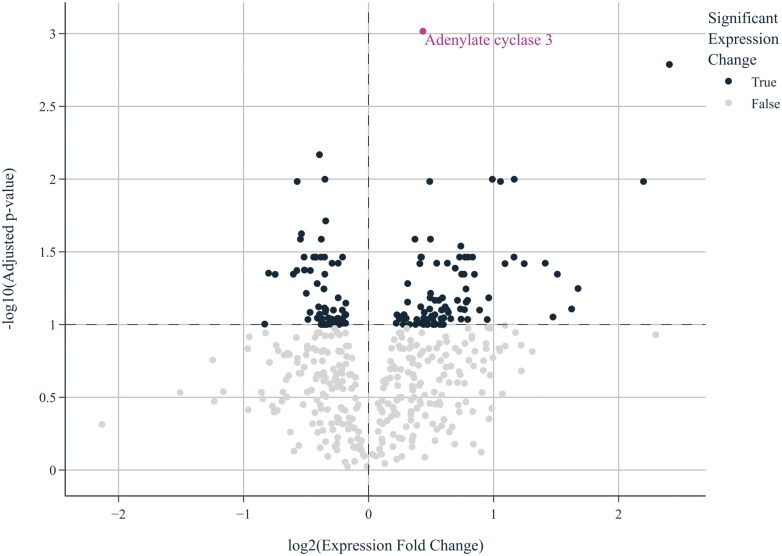
Volcano plot highlighting DEG (identified by black dots) in the muscle tissue of TRT vs. NC pigs (155 DEG; adjusted *P* < 0.1) at 19 wk of age. The differential expression of the adenylate cyclase type 3 gene in is highlighted in the figure.

**Figure 3.**
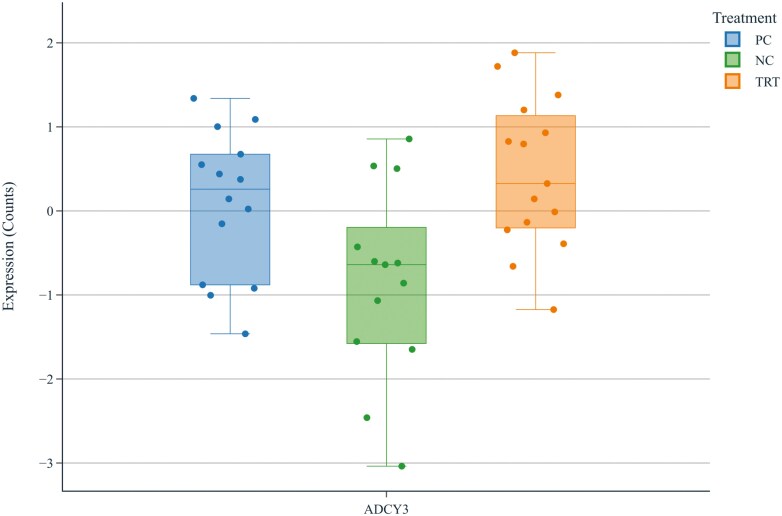
Box plot of Z-normalized log2 abundance of transcripts encoding the ADCY3 gene in the NC, PC, and TRT.

The differential expression of genes in the muscle tissue of pigs in PC vs. NC and in TRT vs. NC is shown in Figures 1 and 2, respectively, via volcano plots. There were 71 DEG in the PC compared to the NC and 155 DEG in TRT vs. NC (adjusted *P* < 0.10). Among the DEG in TRT vs. NC, the greatest difference in expression was observed in the adenylate cyclase 3 gene (ADCY3). A box plot illustrating the Z-normalized log2 abundance of RNA transcripts encoding the ADCY3 gene in the PC, NC, and TRT diets is shown in Figure 3; ADCY3 was differentially expressed in the TRT compared to the NC (adjusted *P* = 0.001, log_2_-fold change 0.43) but not in the PC compared to the NC (adjusted *P* = 0.147, log_2_-fold change 0.24).

The biological pathways that were implicated from the QPA methodology as having been activated or inhibited (based on gene expression in muscle at 19 wk of age) are shown in Table 5 for the PC vs. NC and Table 6 for the TRT vs. NC. In these tables, a positive mean activation score indicates that a given pathway was activated, whereas a negative mean activation score indicates that it was inhibited (relative to the comparator treatment). In the PC vs. NC (Table 5), pathways that were inhibited (adjusted *P* < 0.05) related to arachidonic acid metabolism, biological oxidations, metabolism of water-soluble vitamins, phase II conjugation of compounds, glutathione synthesis and recycling, several hormonal regulation pathways and signal transduction pathways, whereas pathways that were activated (adjusted *P* < 0.05) related to phosphatidylinositol (PI) metabolism, the synthesis of phosphatidylinositol phosphates (PIPs) at the plasma membrane, regulation of insulin-like growth factor (IGF) transport and uptake, death receptor signaling and the mitotic G2-G2/M phases. In the TRT vs. NC (Table 6), pathways that were inhibited (adjusted *P* < 0.05) included phospholipid metabolism, vitamin B1 metabolism, synthesis of inositol phosphate (IP) 3 and 4, phase II—conjugation of compounds, biological oxidations as well as several pathways related to cellular responses to stimuli, hormonal regulation, signal transduction, and the mitotic cell cycle. Pathways that were activated (adjusted *P* < 0.05) included arachidonic acid metabolism, bile acid and bile salt metabolism, retinoid metabolism and transport, cytoprotection by HMOX1, regulation of IGF transport and uptake, insulin receptor recycling, the synthesis of prostaglandins and thromboxanes, and several pathways related to signal transduction and the mitotic cell cycle.

**Table 5.** Mean pathway activation scores for those biological pathways that were identified as being significantly (adjusted *P* < 0.05) activated or inhibited in the PC relative to the NC, as determined by the analysis of gene expression in the muscle of pigs at 19 wk of age using Quantitative Pathway Activation methodology

Pathway	Mean activation score^*a*^	*P*-value^*b*^
Metabolism		
Arachidonic acid metabolism	−1.56	<0.001
Biological oxidations	−1.56	<0.001
Metabolism of water-soluble vitamins and cofactors	−1.31	<0.001
Phase II—conjugation of compounds	−1.20	0.001
Phosphatidylinositol metabolism	1.16	<0.001
Synthesis of PIPs at the plasma membrane	1.17	<0.001
Cellular responses to stimuli		
Glutathione synthesis and recycling	−1.20	0.005
Hormonal regulation		
HSP90 chaperone cycle for steroid hormone receptors (SHR) in the presence of ligand	−2.03	<0.001
Synthesis of prostaglandins and thromboxanes	−1.56	<0.001
ESR-mediated signaling	−1.56	<0.001
Regulation of Insulin-like Growth Factor (IGF) transport and uptake by Insulin-like Growth Factor Binding Proteins (IGFBPs)	1.28	<0.001
Signal transduction		
Signaling by nuclear receptors	−1.56	<0.001
Signaling by FGFR2	−1.50	<0.001
Signaling by WNT	−1.33	<0.001
Death receptor signaling	0.81	0.003
Mitotic cell cycle		
Mitotic G2-G2/M phases	1.22	<0.001

^*a*^Normalized mean pathway activation scores (Z-scores) were calculated based on log_2_-fold changes in the level of gene expression compared to the NC, the level of statistical significance and the topological importance of the expressed genes within a given pathway. Positive values denote a relative activation of a given pathway, and negative values denote a relative inhibition, expressed as the number of standard deviations a given data point lies above or below the mean of the NC treatment.

^*b*^Displayed *P*-values are after adjustment for false discovery rate associated with conducting multiple comparisons, using the Benjamini and Hochberg method (Benjamini and Hochberg, 1995).

**Table 6.** Mean pathway activation scores for those biological pathways that were identified as being significantly (adjusted *P* < 0.05) activated or inhibited in TRT^*a*^ relative to the NC, as determined by the analysis of gene expression in the muscle of pigs at 19 wk of age using a Quantitative Pathway Activation methodology

Pathway	Mean activation score^*b*^	*P*-value^*c*^
Metabolism		
Phospholipid metabolism	−2.09	<0.001
Vitamin B1 (thiamin) metabolism	−1.91	<0.001
Synthesis of IP_3_ and IP_4_ in the cytosol	−1.78	<0.001
Phase II—Conjugation of compounds	−1.77	<0.001
Biological oxidations	−1.60	<0.001
Arachidonic acid metabolism	1.43	<0.001
Bile acid and bile salt metabolism	1.43	<0.001
Retinoid metabolism and transport	1.47	<0.001
Cellular responses to stimuli		
Glutathione synthesis and recycling	−1.77	<0.001
Heme signalling	−1.69	<0.001
Oxidative stress-induced senescence	−1.63	<0.001
Regulation of HSF1-mediated heat shock response	−1.59	<0.001
Cytoprotection by HMOX1	1.12	<0.001
Hormonal regulation		
ESR-mediated signaling	−1.92	<0.001
Estrogen-dependent gene expression	−1.82	<0.001
Estrogen-dependent nuclear events downstream of ESR-membrane signaling	−1.69	<0.001
Regulation of IGF transport and uptake by IGFBPs	1.07	<0.001
Insulin receptor recycling	1.42	<0.001
Synthesis of Prostaglandins and Thromboxanes	1.43	<0.001
Signal transduction		
Signaling by Nuclear Receptors	−1.87	<0.001
Signaling by FGFR2	−1.82	<0.001
MAPK6/MAPK4 signaling	−1.69	<0.001
p38MAPK events	−1.63	<0.001
VEGFA-VEGFR2 pathway	−1.60	<0.001
Signaling by Rho GTPases	0.83	0.006
Signaling by WNT	0.97	0.001
VEGFR2 mediated vascular permeability	1.13	<0.001
Death receptor signaling	1.19	<0.001
p75 NTR receptor-mediated signaling	1.29	<0.001
Amino acids regulate mTORC1	1.41	<0.001
PKA-mediated phosphorylation of CREB	2.22	<0.001
Hedgehog “off” state	2.29	<0.001
Signaling by GPCR	2.34	<0.001
Signaling by hedgehog	2.35	<0.001
Mitotic cell cycle		
M phase	−1.76	<0.001
Mitotic G2-G2/M phases	−1.69	<0.001
Mitotic G1 phase and G1/S transition	1.65	<0.001
S phase	1.75	<0.001

^*a*^TRT = NC + Maxificient Boost GF, a combination of 25(OH)D_3_ plus triterpenoids (500 mg/kg; MaxiFicient Boost GF, DSM-Firmenich, Switzerland corresponding to 1,500 IU/kg vitamin D).

^*b*^Normalized pathway activation scores (*Z*-scores) were calculated based on log2fold changes in the level of gene expression compared to the NC, the level of statistical significance and the topological importance of the expressed genes within a given pathway. Positive values denote a relative activation of a given pathway, and negative values denote a relative inhibition, expressed as the number of standard deviations a given data point lies above or below the mean of the NC treatment.

^*c*^Displayed *P*-values are after adjustment for false discovery rate associated with conducting multiple comparisons, using the Benjamini and Hochberg method (Benjamini and Hochberg, 1995).

## DISCUSSION

Calcium and P are essential for multiple physiological roles in the body, including growth, development, and maintenance of the skeletal system. The homeostatic control of the two minerals is closely interrelated such that a deficit or excess of either can affect the absorption and utilization of the other. In grower-finisher pigs, if the dietary Ca content or Ca:P ratio is inadequate, there is a potential for reduced bone mineralization (Stein, 2023). This is a particular risk during the finisher phase (75–135 kg BW) because daily protein synthesis is low, and most of the absorbed Ca and P is used for bone synthesis as opposed to muscle growth (Lee et al., 2023). Conversely, if Ca is in excess, especially if the STTD of P in the diet is at or below the requirement, there is a potential for restricted growth. However, pigs may tolerate excess Ca if P is provided above the requirement (Lee et al., 2023). In the present study, the PC and NC diets were both formulated with levels of STTD P (0.29% and 0.26%, respectively) that were approximated at NRC requirement level (0.31%, 0.27%, and 0.24% for 25–50 kg BW, 50–75 kg BW, and 75–100 kg BW, respectively; NRC, 2012) and both contained supplementary phytase which was expected to release additional iP from the diet. On the other hand, Ca was above NRC requirements in the PC (0.70% vs. NRC requirements of 0.66%, 0.55%, and 0.52%, respectively, for 25–50 kg BW, 50–75 kg BW, and 75–100 kg BW) and below NRC requirements in the NC (0.49%). It is therefore consistent with the expectation that there was no significant difference in growth performance between the NC vs. PC over the study period, whereas BMC (based on whole-body scanning or bone ash when expressed in g/bone) was significantly reduced. The latter was likely due to the deficiency of Ca in the NC diet. The increased tissue percentage of pigs fed the NC vs. PC at 19 wk of age is consistent with the reduced bone percentage, although metacarpal bone ash, Ca, and P, when expressed in percentage terms, did not show this same difference. This could be explained by the fact that reduced bone mineralization in the NC vs. PC reduces the size of the entire bone, which in turn reduces the weight of Ca and P in the bone without altering their percentages in the bone ash.

No reductions in serum Ca or P or markers of bone formation (osteoclastin and alkaline phosphatase) or resorption (CTX) were evident in NC vs. PC pigs. Other studies have noted reduced serum content of Ca and P in younger pigs fed P and Ca-deficient diets (Zhou et al., 2022). The absence of a difference in plasma P content between the NC and PC may have been because levels of STTD of P in the diet were not that different between the two treatments (0.26% vs. 0.29% in the diet) and the addition of the phytase would have released additional iP into the circulation which would have raised available P above these levels. On the other hand, the absence of a difference in plasma Ca could be due to the fact that there are multiple Ca-sensing receptors distributed in the body (Votterl et al., 2021).

It is common practice to use vitamin D status as a marker of the effect of dietary interventions on bone mineralization in pigs due to its critical role in the absorption and retention of Ca and P (O’Doherty et al., 2010). Vitamin D status is primarily assessed by measuring circulating levels of 25(OH)D_3_, this being the tissue storage form of vitamin D that has already been hydroxylated in the liver and can later be further hydroxylated to the metabolically active form, 1,25-(OH)_2_D_3_ in the kidney, for use as needed. Currently used reference values for circulating 25(OH)D_3_ lie between 18 and 30 ng/mL according to Madson et al. (2012), meaning that there is a known risk for reduced bone mineralization if circulating levels fall below 18 ng/mL. However, more recent research by Williams et al. (2024) has questioned the applicability of these values to the general pig population and instead reported mean concentrations of 25(OH)D_3_ in the serum of pigs classified as healthy, lame, and unhealthy, of 25, 21, and 16 ng/mL, respectively. Meanwhile, in humans, a retrospective study of more than 10,000 observations concluded that the clinical cutoff for sufficient circulating 25(OH)D_3_ level is 30 ng/mL (75 nmol/L) based on PTH hormone levels (Serdar et al., 2017). These data suggest that levels of circulating 25(OH)D_3_ below 30 ng/mL could be insufficient for healthy Ca and P metabolism.

In the present study, the analyzed concentrations of Vitamin D_3_ and 25(OH)D_3_ in plasma at 19 wk of age confirmed the presence of vitamin D_3_ in the circulation of pigs offered the PC and NC diets and its absence in pigs fed the TRT diet. This was as expected based on the diet compositions. We observed no difference in plasma 25(OH)D_3_ concentrations of pigs offered the NC and PC, also as expected. The values obtained in these treatments (20.14 and 19.10 and ng/mL, respectively), were in the lower part of the normal range according to Madson et al. (2012) and similar than those of pigs classified as lame by Williams et al. (2024). Pigs offered the TRT diet, which included 25(OH)D_3_ as part of the combination, showed substantially higher levels of 25(OH)D_3_ (53.1 ng/mL) than pigs offered the NC or PC diets. The biological implications of a high vitamin D status (more than 30 ng/mL) are not clear from the wider literature. In humans, observational studies have suggested that circulating 25(OH)D_3_ levels between 40–60 ng/mL can protect against viral infection from COVID-19 by activating the immune system (Bae et al., 2022), and that levels between 50 and 125 ng/mL may benefit athlete performance and the prevention of injuries at muscle level (de la Puente Yagüe et al., 2020). Interestingly, we additionally measured levels of 24,25-(OH)_2_-D_3_ in plasma and observed that these were also significantly elevated in TRT vs. NC or PC. The 24,25-(OH)_2_-D_3_ form of vitamin D_3_ has been described as an inactivated form that is produced when circulating vitamin D_3_ is surplus to requirements (Hasan et al., 2023). Its increase in TRT could therefore indicate that vitamin D levels were slightly higher than required. However, this is not necessarily the case because other research has questioned whether 24,25-(OH)_2_-D_3_ is a waste product, instead suggesting it may have a functional role in fracture repair (Martineau et al., 2018).

The absence of a difference in the level of Ca in the plasma across treatments suggests that homeostatic mechanisms regulating Ca in circulation were operating efficiently to compensate for the dietary differences in Ca content. However, plasma P concentration and CTX, an indicator of bone resorption, were both higher in pigs that offered TRT than NC. This suggests a compensatory effect of the combination of 25(OH)D_3_ with triterpenoids, likely from the supplemented vitamin D (in the form of 25(OH)D_3_), on mineral status in pigs fed the Ca-deficient diet. The bone quality results (BMD and BMC based on whole-body scanning) at 19 wk of age are consistent with this and suggest that pigs in TRT were able to fully compensate for the negative effect of the reduction in dietary Ca on bone mineralization that was evident in the NC, bringing BMD and BMC up to a level not different from that achieved by the Ca-adequate PC diet. In weaned pigs, Zhang et al. (2022) observed a similar compensatory effect of supplemental 25(OH)D_3_ over vitamin D_3_ on mineral status. In these piglets fed low Ca and P diets, increased serum Ca concentration rather than increased P concentration was reported, whereas Zhou et al. (2022) showed a beneficial effect of 25(OH)D_3_ (over vitamin D_3_) on both serum and bone Ca and P levels as well as Ca and P apparent digestibility and retention. Studies in growing-finishing pigs are limited, and results have been inconsistent. Duffy et al. (2018) reported no beneficial effect on metacarpal bone parameters of 25(OH)D_3_ vs. vitamin D_3_-supplementation to low-P diets containing phytase in pigs of 60 to ~110 kg BW. In contrast, Zhou et al. (2023) observed increased bone-breaking strength, bone Ca and P percentage and increased relative mRNA expression of Ca channels in the liver of 45 to ~120 kg pigs fed P and Ca adequate diets supplemented with 25(OH)D_3_, but only when the additive was supplemented in nursery phase as well as the grower and finisher phases. The present findings contradict these previous studies by showing that supplementation of the additive combination containing 25(OH)D_3_ to a Ca-deficient, P-adequate diet containing phytase, during grower-finisher phase only, improved bone mineralization to a level equivalent to that produced by a Ca- and P-adequate diet containing vitamin D_3_ (and phytase). Results suggest that the dietary combination of 25(OH)D3 with triterpenoids could be useful for maintaining healthy bone composition in grower-finishing pigs fed Ca- and P-reduced diets, together with phytase supplementation.

Alongside the improvements in bone mineralization, the dietary combination of 25(OH)D3 with triterpenoids in TRT also increased BW and ADG during the overall (grower-finisher) period (vs. the NC). This indicated a direct effect of the combination of 25(OH)D_3_ and triterpenoids on tissue deposition and growth that was superior to the effect of supplementation with D_3_ (via the NC). The effect was largely evident during the finisher phase (16–19 wk of age) when growth rates were high, but Ca and P requirements for muscle synthesis were lower (relative to other phases). Feed intake over the experimental period was increased alongside ADG, but to a lesser extent in percentage terms, which may suggest improved efficiency of conversion of feed nutrients into growth, although this was not evident from measurements of FCR. The improved BW and ADG are in contrast to the findings of the study by Zhou et al. (2023), who observed no effect of 25(OH)D_3_ supplementation during grower-finisher phase only (50 kg to market weight) on growth performance compared with supplementation of vitamin D_3_ alone at equivalent dose. The study by Duffy et al. (2018) also reported no effect of 25(OH)D_3_ (with or without phytase) on the growth performance of finisher pigs. The reason for the different results may be related to the Ca and P-adequacy of the basal diets. Those used by Zhou et al. (2023) were adequate in both minerals and by Duffy et al. (2018) were deficient in P but not Ca. This may have meant that mineral balance in these studies was more optimal for growth performance regardless of 25(OH)D_3_ supplementation. Alternatively (or in addition), the presence of the triterpenoids in the additive combination used in the present study could have enhanced the beneficial effect on the growth performance of TRT-supplemented pigs via its antioxidant and antiinflammatory effects. In particular, triterpenoids can have a strong protective effect against skeletal muscle atrophy (Yadav et al., 2022). Growth performance responses (all measures, all timepoints) in the TRT diet were consistently at the same level or improved compared to the PC, suggesting that the additive combination fully compensated for the 30% reduction in Ca and 10% reduction in STTD of P applied to the NC diet. This effect was over and above any effect from the phytase, which was present at 1,000 FYT/kg in all diets.

The analysis of the relative expression of different genes in the *Longissimus dorsi* muscle of pigs in TRT compared with the NC at the end of the experiment (19 wk of age) can be used to explain the beneficial effects of the dietary combination of 25(OH)D3 with triterpenoids on growth performance. A major pathway modulated in TRT vs. NC pigs was signal transduction. Several of the activated signal transduction pathways, including p75 NTR receptor-mediated signaling and hedgehog, are known to play roles in muscle development and repair (Deponti et al., 2009; Peterson and Henry, 2010). In both the PC and TRT relative to the NC, the regulation of IGF transport and uptake was also activated, accompanied by activation of phosphatidyl inositol metabolism and synthesis of PIPs at the plasma membrane and by insulin receptor recycling and inhibition in the synthesis of inositol triphosphate (IP3) and IP4. Insulin and IGF1 bind to receptors that activate a cascade of phosphorylation events, resulting in the enhancement of protein synthesis and inhibition of protein breakdown in muscle through the activation of mTOR (Sartori et al., 2021); IP3 and IP4 are also involved in muscle cell functioning, specifically Ca transport into muscle cells (Berridge, 2016). It is commonly reported that specific triterpenoids have beneficial effects on insulin signaling with the potential to remediate insulin resistance and increase glucose uptake into cells (Lee et al., 2021; Wei et al., 2022). There was also significant activation of the regulation of the mTOR Complex 1 (mTORC1) by the amino acids pathway in TRT relative to the NC. The mTORC1 is a key pathway governing muscle growth that mediates the anabolic response to nutrients, insulin, IGF, and exercise (Mounier et al., 2011). Considering that animals with impaired mTORC1 signaling show decreased muscle mass (Mounier et al., 2011), the activation of this pathway in TRT is predicted to have been associated with the observed increased growth performance of pigs in TRT relative to the NC. Although a positive effect of 25(OH)D_3_ on muscle growth has previously been described in relation to the supplementation of sows and the development of primary muscle fibers in their fetuses (Hines et al., 2013), this is the first report of positive muscle growth effects in older, growing-fattening pigs. Our findings provide insight into the mechanism of this effect when 25(OH)D_3_ is combined with triterpenoids, implicating mTOR signaling. This is consistent with the implications of similar research in broilers; Vignale et al. (2015) demonstrated that 25(OH)D_3_-supplemented broilers exhibited enhanced breast meat yield, protein synthesis, and expression of vitamin D receptor (VDR) in muscle, and (through in vitro study) that 25(OH)D_3_ activated the mTOR/S6 kinase pathway in quail myoblast (QM7) cells.

Another pathway activated in TRT vs. NC of relevance to muscle growth was cAMP-dependent protein kinase (PKA)-mediated phosphorylation of cAMP response element-binding protein (CREB). Activation of CREB is via cAMP through direct PKA-mediated phosphorylation in response to acute exercise or stress (Berdeaux and Hutchins, 2019). Activation of PKA induces muscle protein synthesis, whereas cAMP inhibits muscle atrophy, increasing muscle size and strength (Berdeaux and Stewart, 2012). This implicates PKA and CREB signaling in the improved weight gain of TRT vs. NC pigs. The greater abundance of transcripts encoding ADCY3, which initiates cAMP signaling in the membrane of skeletal muscle fibers (Berdeaux and Stewart, 2012) in TRT vs. NC, is consistent with this. A relationship between activation of PKA through cAMP and activation of mTORC1 has previously been reported in the scientific literature (Soliman et al., 2010; Liu et al., 2016; Melick and Jewell, 2020), and several different triterpenoids have been shown to directly activate CREB (Yadav et al., 2010). It was also interesting that the metabolism of arachidonic acid and WNT signaling were inhibited in the PC relative to NC but activated in TRT relative to NC, alongside the activation of prostaglandin synthesis. Arachidonic acids are used to synthesize eicosanoids, which have an important role in the innate immune response (Funk, 2001). One such eicosanoid group is the prostaglandins, which have a role in promoting inflammation but also affect WNT signaling (Yui et al., 2015). Interestingly, WNT signaling can stimulate mTOR signaling by inhibiting GSK3 phosphorylation of TSC2 (Melick and Jewell, 2020). Hence, activation of WNT in TRT pigs could have been another route by which mTOR was activated to enhance muscle growth.

In conclusion, in grower-finisher pigs, supplementation of 25(OH)D_3_ in combination with triterpenoids to a Ca-deficient, P-adequate diet containing phytase, increased circulating vitamin D status, improved growth performance, whole-body bone deposition and mineralization, plasma P and metacarpal Ca and P content (g/bone basis) compared to supplementation of classical vitamin D_3_ at an equivalent dose level. Further, the dietary combination of 25(OH)D_3_ with triterpenoids led to differential expression of genes in muscle tissue, which indicated the activation of several biological pathways involved in the activation of muscle growth and the inhibition of muscle atrophy, including pathways that activate mTOR, a key central regulator of cell metabolism, growth, proliferation, and survival. Overall growth performance responses of pigs offered the dietary combination of 25(OH)D_3_ with triterpenoids supplemented, Ca-deficient, P-adequate diet were not different to those of pigs fed an unsupplemented diet adequate in both Ca and P. Collectively, the results suggest that the dietary combination of 25(OH)D_3_ with triterpenoids has potential for use, in synergy with phytase, in supporting a reduction in Ca and P in the diet of grower-finishers to reduce nutrient waste and improve the sustainability of pig production.
